# Eradication of multidrug-resistant *Acinetobacter baumannii *in a female patient with total hip arthroplasty, with debridement and retention: a case report

**DOI:** 10.1186/1752-1947-3-45

**Published:** 2009-02-03

**Authors:** Alison M Beieler, Robert W Belknap, Michael R Dayton, Connie S Price, Steve J Morgan

**Affiliations:** 1 Department of Medicine, Infectious Diseases, Denver Health Medical Center, 660 Bannock St, Denver, CO 80204, USA; 2 Department of Medicine & Public Health, Infectious Diseases, Denver Health Medical Center, 605 Bannock St, Denver, CO 80204, USA; 3 Department of Orthopedics, University of Colorado, 1635 N. Ursula St, Aurora, CO 80045, USA; 4 Department of Orthopedics, Denver Health Medical Center, 777 Bannock St, Denver, CO 80204, USA

## Abstract

**Introduction:**

Multidrug-resistant *Acinetobacter baumannii *has become a significant cause of healthcare-associated infections, but few reports have addressed *Acinetobacter baumannii *infections associated with orthopedic devices. The current recommended treatment for complicated infections due to orthopedic devices, including resistant gram-negative rods, consists of antimicrobial therapy with debridement and removal of implants.

**Case presentation:**

The patient, a 47-year-old woman, had previously had a prior total hip arthroplasty at 16 years of age for a complex femoral neck fracture, and multiple subsequent revisions. This time, she underwent a fifth revision secondary to pain. Surgery was complicated by hypotension resulting in transfer to the intensive care unit and prolonged respiratory failure. She received peri-operative cefazolin but postoperatively developed surgical wound drainage requiring debridement of a hematoma. Cultures of this grew ampicillin-sensitive *Enterococcus *and *Acinetobacter baumannii *(sensitive only to amikacin and imipenem). The patient was started on imipenem. Removal of the total hip arthroplasty was not recommended because of the recent surgical complications, and the patient was eventually discharged home. She was seen weekly for laboratory tests and examinations and, after 4 months of therapy, the imipenem was discontinued. She did well clinically for 7 months before recurrent pain led to removal of the total hip arthroplasty. Intra-operative cultures grew ampicillin-sensitive *Enterococcus *and coagulase-negative *Staphylococcus *but no multidrug-resistant *Acinetobacter baumannii*. The patient received ampicillin for 8 weeks and had not had recurrent infection at the time of writing, 37 months after discontinuing imipenem.

**Conclusion:**

We describe the successful treatment of an acute infection from multidrug-resistant *Acinetobacter baumannii *with debridement and retention of the total hip arthroplasty, using monotherapy with imipenem. This case challenges the general assumption that all orthopedic-device infections due to multidrug-resistant gram-negative organisms will require hardware removal. Further studies are needed to determine if organisms such as multidrug-resistant *Acinetobacter baumannii *are amenable to treatment with hardware retention.

## Introduction

*Acinetobacter baumannii *has become an important cause of healthcare-associated infections worldwide [1]. The organism has developed substantial antimicrobial resistance, making treatment of infections attributed to *A. baumannii *increasingly more difficult to manage. Multidrug-resistant (MDR) *A. baumannii *is capable of causing a variety of infections, including urinary tract infections, pneumonia, bacteremia, as well as skin and soft tissue infections [2]. It is found primarily in patients with prolonged stays in intensive care units and less commonly in outpatients [3].

*A. baumannii *has been reported with increasing frequency as a cause of device-associated infections, related to catheters and mechanical ventilation [1,2]. Most recently, MDR *A. baumannii *has been described as a cause of osteomyelitis in soldiers of the Iraq war [4] and in trauma patients [5].

Despite its increasing frequency, there is little data in the literature on the treatment of orthopedic-device infections due to MDR *A. baumannii*, specifically on total joint arthroplasties. The recently recommended approach for treating infected arthroplasties is a two-step hardware exchange with use of an antibiotic cement spacer (PROSTALAC) [6]. Here, however, we describe the eradication of MDR *A. baumannii *total joint arthroplasty infection in a patient using a debridement and retention approach with imipenem-cilastatin (imipenem) monotherapy.

## Case presentation

A 47-year-old woman was involved in a motor vehicle accident at the age of 16 and suffered a right femoral neck fracture. Open reduction internal fixation of the fracture was not possible, and the primary therapy for the injury was a total hip arthroplasty (THA). The patient required four revisions of her primary total hip. She presented to our center for consideration for a fifth revision surgery secondary to intractable pain. She was taken to the operating room (OR) and received peri-operative cefazolin as per protocol. Complications arose during the revision procedure. A femoral shaft fracture occurred, and a deep pelvic arterial injury resulted in intra-operative hemodynamic instability, requiring suspension of the procedure and arterial embolization. The patient was subsequently transferred to the intensive care unit and, on day 2 in hospital, returned to the OR for completion of the original procedure which was uneventful. Her postoperative course, however, was complicated by prolonged respiratory failure necessitating a tracheostomy.

On day 12 in hospital, the patient developed increased pain, redness and new drainage from her surgical wound. She underwent surgical exploration, was found to have a hematoma with evidence of defects in the deep fascia, which required debridement down to the hip prosthesis. The hematoma was evacuated, no antibiotic beads were placed, and she was started on vancomycin. Quantitative cultures were not performed; however, tissue cultures grew ampicillin-sensitive *Enterococcus *species and *A. baumannii *(sensitive only to amikacin and imipenem). The patient was switched to imipenem 500 mg IV every 6 hours on day 15 in hospital. She was not considered to be a candidate for removal of her prosthesis because of the recent prior surgical complications.

On day 33 in hospital, the patient returned to the OR for debridement because of continued fevers despite imipenem; cultures once again grew MDR *A. baumannii*, *Enterococcus *species and coagulase-negative *Staphylococci *(CNS). The patient improved and was discharged home on day 53 to complete her imipenem therapy at home.

The patient was seen weekly in our Orthopedic Infectious Diseases Clinic for blood tests and clinical examination. Due to persistently elevated inflammatory markers, erythrocyte sedimentation rate (ESR) and C-reactive protein (CRP), her antibiotic course was extended. Her ESR and CRP improved but never normalized. The antibiotics were stopped after completing a 4-month course of imipenem. The patient was not placed on oral suppressive therapy since no options existed for the MDR *A. baumannii*.

The patient remained symptom-free for 7 months before noticing pain with walking and swelling at the right hip. She denied having had any new injury or trauma. On exam, she had redness, warmth, swelling and new drainage at the incision site on the right hip, presumed to represent recurrence of her MDR *A. baumannii *infection. At this time, she underwent surgical debridement and removal of the THA. Intra-operative cultures were obtained but the prosthesis was not sonicated. The operative cultures grew *Enterococcus *species (sensitive to ampicillin and penicillin), and two types of CNS (resistant to penicillin), which were not thought to be pathogenic. There was no MDR *A. baumannii *isolated. The patient responded to an 8-week course of therapy directed only toward the *Enterococcus *species with ampicillin. Twenty-nine months after stopping antibiotics, she remains free of infection at the time of writing.

## Discussion

*Acinetobacter *is an aerobic gram-negative coccobacilli that is catalase positive, oxidase negative, non-fermenting and nonmotile [2]. Although it is usually a low-grade pathogen, it has several properties that can increase its virulence. These include a capsule made of polysaccharide (making the organism more hydrophilic), the capability of adhesion to epithelial cells in the company of capsular polysaccharide, enzyme production which can injure lipid components of tissue, and possibly a toxic-type function found in the cell wall lipopolysaccharide [2]. *Acinetobacter *is typically found in environmental areas such as soil and aquatic areas [7]; thus, it can often be a contaminant associated with the environment. However, *A. baumannii *has recently been linked to hospital infections, including surgical wound infections and device-related infections [1,2].

In a healthcare setting, patients become colonized with the organism on their skin, usually in moist places, the toe webs, axillae and groin [2]. *Acinetobacter *can live in the hospital setting for several days; it has been demonstrated to survive on filter paper for up to 6 days, on Formica for 13 days and on cotton for 25 days [8]. Risk factors for acquisition of MDR *A. baumannii *during outbreaks have been described and include the use of broad-spectrum antibiotics, longer hospitalization, advanced age, mechanical ventilation, trauma intensive care unit and the use of pulsatile lavage wound irrigation [2,9].

Before the year 1970, most *Acinetobacter *species were sensitive to carbenicillin, ampicillin, gentamicin, or minocycline [2]. Around 1975, *Acinetobacter *was documented as developing resistance to many antibiotics including tetracyclines, cephalosporins, cephamycins, aminopenicillins and aminoglycosides [2]. MDR *A. baumannii *can be defined as an organism resistant to over three classes of antibiotics [4]. Some *A. baumannii *are sensitive to newer cephalosporins (ceftazidime), quinolones, amikacin, tobramycin and imipenem, but often with higher minimum inhibitory concentrations than before [2].

Recently, increases in imipenem-resistant MDR *A. baumannii *infections have lead to the use of colistin or polymyxin B for salvage therapy in severe cases [10]. Colistin and polymyxin B have proven to be successful treatments of MDR *A. baumannii *bacteremia, pneumonia, meningitis and orthopedic-device infections in documented case reports [10].

Recent cases of MDR *A. baumannii *infections in military personnel have occurred in the form of bacteremia, extremity war wounds and osteomyelitis [4]. In these cases, osteomyelitis was documented via bone cultures taken intra-operatively during open debridements or internal/external fixation procedures [4]. Other patients with symptoms of fever, increased white blood cell count, open fractures with necrotic tissue and gross pus with deep tissue cultures positive for MDR *A. baumannii *were also classified as osteomyelitis [4]. In the military patients treated for osteomyelitis, ampicillin/sulbactam, ceftazidime and amikacin in combination with imipenem were used successfully [4]. The duration of therapy in this small study of 18 patients varied between 4 to 8 weeks of IV treatment [4]. In a mean follow-up time of 9 months, there were no recurrences of MDR *A. baumannii *infection [4].

Previously published guidelines related to orthopedic device infections suggest that prerequisites for debridement and retention include short duration of symptoms, satisfactory condition of soft tissue and the absence of difficult-to-treat resistant microorganisms [6], particularly gram-negative organisms [11]. These guidelines, as they relate to gram-negative pathogens such as *A. baumannii *as well as MDR phenotypes, are based on little evidence and mostly on speculation. This is due, in part, to the inability to confirm microbiologic eradication of the offending pathogen. In our patient's case, microbiologic eradication was documented in a subsequent debridement several months after antibiotics had been discontinued.

New information suggests that *A. baumannii *is capable of forming biofilms on different surfaces, which may explain in part why these infections are difficult to treat. *A. baumannii *biofilms that adhere to various glass (hydrophilic) and plastic (hydrophobic) surfaces were observed in recent studies [12]. After allowing the organism to set for approximately 10 to 15 minutes, the biofilm made by the *A. baumannii *19606 strain was shown to remain on both Teflon strips and Petri dishes after washing with tap water [12]. The *A. baumannii *biofilm demonstrated growth in both static and dynamic environments and at varying temperatures, verifying that an *A. baumannii *organism can persevere and multiply in harsh conditions [12]. This information suggests that device-related infections due to *A. baumannii *require definitive antibiotic therapy and, if treatment fails to be effective, possible device removal for cure.

## Conclusion

*A. baumannii *continues to emerge as an important cause of trauma-associated infections and orthopedic device-associated infections. Consequently, more evidence is necessary to understand the importance of gram-negative infections and MDR phenotypes, especially MDR *A. baumannii*, and factors associated with failure when salvage is attempted. It is interesting that the more sensitive gram-positive organism (ampicillin-sensitive *Enterococcus*) persisted in our patient despite active therapy against this organism, again calling into question the assumption that gram-negative organisms are inherently more difficult to eradicate.

We described a case report of successful treatment of a MDR *A. baumannii *infected THA with debridement and retention, and 4 months of monotherapy using imipenem, with microbiologic eradication of MDR *A. baumannii*, and no signs of recurrence with this organism at 37 months. Assumptions that orthopedic device infections due to gram-negative organisms and/or those with multidrug-resistant phenotypes are not amenable to salvage should be re-examined.

## Abbreviations

MDR: Multidrug-resistant; THA: total hip arthroplasty; OR: operating room; CNS: coagulase-negative *Staphylococcus*; ESR: erythrocyte sedimentation rate; CRP: C-reactive protein.

## Consent

The patient gave verbal consent to have her case written up for a published medical report. Due to mobility issues the patient preferred to give verbal consent for this case presentation. Documentation of this consent is available for review by the Editor-in-Chief of this journal.

## Competing interests

The authors declare that they have no competing interests.

## Authors' contributions

AMB was the provider who assisted in caring for the patient while on antibiotic therapy, obtained consent, obtained chart records, prepared the manuscript, and submitted the manuscript for publication. RWB was the Infectious Disease specialist who assisted in caring for the patient, and contributed to manuscript editing for medical accuracy and content. MRD was the Orthopedic surgeon caring for the patient, and assisted with manuscript editing for medical accuracy. CSP was the Infectious Disease specialist who assisted in caring for the patient and contributed to manuscript editing for medical accuracy and content. SJM was the Orthopedic surgeon caring for the patient, and assisted with collection of chart records, and manuscript editing for medical accuracy.
